# GC-MS Composition and Olfactory Profile of Concretes from the Flowers of Four *Nicotiana* Species

**DOI:** 10.3390/molecules25112617

**Published:** 2020-06-04

**Authors:** Venelina Popova, Tanya Ivanova, Albena Stoyanova, Violeta Nikolova, Tsveta Hristeva, Valtcho D. Zheljazkov

**Affiliations:** 1 Department of Tobacco, Sugar, Vegetable and Essential Oils, University of Food Technologies, 4002 Plovdiv, Bulgaria; vpopova2000@abv.bg (V.P.); tantonieva@mail.bg (T.I.); aastst@abv.bg (A.S.); 2 Tobacco and Tobacco Products Institute–Bulgarian Agricultural Academy, 4108 Markovo, Bulgaria; nikolova.v@abv.bg (V.N.); zveta_h@abv.bg (T.H.); 3 Department of Crop and Soil Science, Oregon State University, Corvallis, OR 97331, USA

**Keywords:** aromatic products, concretes, *N. rustica*, *N. alata*, *N. glutinosa*, *N. tabacum*

## Abstract

The genus *Nicotiana* (Solanaceae) includes over 70 species, with a long history of traditional use; many of them are nowadays used in bioengineering, biosynthesis, molecular biology, and other studies, while common tobacco, *N. tabacum* L., is one of the most economically important industrial crops worldwide. Although *Nicotiana* species have been extensively investigated, relatively less research has focused on flowers, especially research related to obtaining aromatic products for cosmetic and perfumery use. On the other hand, there is evidence that *Nicotiana* flowers accumulate various secondary metabolites with a distinct aroma and biological activities, and the flowers represent a biomass available in sufficient quantities. Therefore, this study aimed to determinate the chemical composition (by GC-MS) and the olfactory profiles of a specific type of natural aromatic product (concrete), obtained from the flowers of four *Nicotiana* species, in a direct comparison between them. The yields of extracted concrete were sufficiently high, varying between the species, 1.4% (*N. rustica* L.), 2.5% (*N. glutinosa* L.), 1.6% (*N. alata* Link&Otto genotype with white flowers), 2.7% (*N. alata* genotype with pink flowers), 3.2% (*N. tabacum*, Oriental type), and 5.2% (*N. tabacum*, Virginia type). The major components of the obtained concretes belonged to different chemical classes: *N. rustica* and *N. tabacum* (OR), the hydrocarbons *n*-tetratriacontane (14.5%; 15.0%) and *n*-triacontane (12.1%; 13.3%), and 3-methyl-pentanoic acid (11.1%; 12.2%); *N. glutinosa*, the diterpenes sclareol (25.9%), 3-α-hydroxy-manool (16.3%), and 13-epimanool (14.9%); *N. alata* (WF), the phenylpropanoid terephthalic acid and di(2-ethylhexyl) ester (42.9%); *N. alata* (PF), the diterpene tributyl acetylcitrate (30.7%); and *N. tabacum* (FCV), the hydrocarbons *n*-hexacosane (12.9%) and *n*-pentacosane (12.9%). Each of the flower concretes revealed a characteristic odor profile. This is the first report about *Nicotiana* species as a source for obtaining flower concretes; these initial results about the concrete yield, olfactory profile, and chemical composition are a prerequisite for the possible processing of *Nicotiana* flowers into new aromatic products for use in perfumery and cosmetics. The study provides new data in favor of the potential of the four *Nicotiana* species as aromatic plants, as well as a possible alternative use of flowers, a valuable, but discarded, plant material in other applications.

## 1. Introduction

*Nicotiana* is one of the eight largest genera in the family Solanaceae, with 76 naturally occurring species, most of which are indigenous to North and South America and Australia, and also species that originate from Africa [1,2]. According to the sectional classification of the genus, provided in Coodspeed’s monograph [3] and its recent revisions [1,2,4], the four species used in this study belong to the following *Nicotiana* sections: *N. alata* Link&Otto, section *Alatae; N. tabacum* L., section *Nicotiana*; *N. glutinosa* L., section *Undulatae*; and *N. rustica* L., section *Rusticae. Nicotiana* species have a long history of medicinal, recreational, ceremonial, ornamental, and other traditional uses in their countries of origin [5], which was further extended in response to the growing importance of the species. Cultivated tobacco, *N. tabacum* L. (and to a lesser extent *N. rustica* L.), is nowadays one of the most economically important industrial crops worldwide [2]. Also, many species in the genus are widely used as model plants in bioengineering, in plant defense mechanism studies, and in biosynthesis and molecular biology research [2,6].

*Nicotiana* species have been extensively investigated, and the diversity of biologically active specialized (secondary) metabolites synthesized by *Nicotiana* plants has been pointed out as a characteristic trait [2,7]. Although the most extensive research has been directed to the metabolite profiles of common tobacco (*N. tabacum*) leaves, with over 4000 individual compounds reported [8,9], the chemical composition of the flowers of different *Nicotiana* species has also been the subject of scientific interest. A number of studies related the chemistry of flower emitted scent with pollination, revealing the involvement of various classes of plant metabolites [10,11,12,13]. Terpene and phenylpropanoid volatiles, various classes of polyphenols, and other compounds were characterized in several species [6,11,12,13,14,15,16,17,18,19]. Plant defense mechanisms were also related to the transport of aroma substances and alkaloids, naturally synthesized by the respective *Nicotiana* species [20,21]. The circadian rhythm of flower scent emissions issued day/night changes regarding the volatile composition, documented by headspace or other trapping techniques for different species [10,11,22].

Substantial differences, with regard to scent-emitted volatiles, were observed between *Nicotiana* species, as well as between different cultivars within a single species or between plants from different locations. In a study on the enzymatic synthesis of 1,8-cineole to α-terpineol, flowers of *N. alata, N. bonariensis* Lehm., *N. forgetiana* Hemsl., *N. longiflora* Cav., and *N. mutabilis* Stehman&Samir (all of section *Alatae* [2]) were found to contain the monoterpenes 1,8-cineole, limonene, β-myrcene, α- and β-pinene, sabinene, and α-terpineol, with distinct quantitative variations between the species [23,24]. In another study, the major headspace volatiles in the flowers of different species were as follows: *N. tabacum*: caryophyllene (109–870.5 ng/g flower) and linalool (301.8 ng/g); *N. sylvestris* Speg&Comes: caryophyllene (838.5 ng/g) and benzyl alcohol (403.0 ng/g); *N. rustica*: epoxytagetone (36.0 ng/g); *N. suaveolens* Lehm: methyl benzoate (473.1 ng/g) and 1,8-cineole (117.2 ng/g); *N. alata*: 1,8-cineole (50.8 ng/g); and *N. tomentosiformis* Goodsp.: methylpentanol (38.3 ng/g), hexanol (33.5 ng/g), and 4-methylhexanol (27.8 ng/g) [25]. The essential oil of flower buds from flue-cured tobacco (*N. tabacum*) planted in two areas in China differed substantially in chemical composition; the first [26] contained 1H-cycloprop[e]azulene (12.22%), (15,3S)-(+)-m-mentha-4,8-diene (8.36%), terpinolene (6.74%), thunbergol (4.16%), and β-4,8,13-duvatriene-1,3-diol (3.69%) as major constituents, and the second [27]: β-cembrenediol (12.24%), carotol (8.55%), isolimonene (7.37%), thunbergol (4.88%), and 9,12-octadecadienoic acid (Z,Z) (4.09%). The oils had different antimicrobial activities against Gram-positive and Gram-negative bacteria, *Bacillus subtilis, Paenibacillus polymyxa*, and *Escherichia coli*; and fungi, *Aspergillus niger*, *Penicillium glaucum*, and *Pichia pastoris* [26,27]. Both oils revealed potent OH and O_2_ radical scavenging effects.

Relatively less studies have been conducted on extracts from the flowers of *Nicotiana* species. The major components extracted from the flowers of *N. longiflora* were manool (21.2%), neophytadiene (13.0%), 2,4-diphenyl-4-methyl-2(*Z*)pentene (8.6%), and (*Z*)-3-hexenol (4.9%) [28]; in turn, the major components for *N. forgetiana* were pentacosane (8.4%), tricosane (8.2%), (*Z*)-3-hexenol (7.8%), heneicosane (6.1%), farnesyl acetone (4.97), ꞵ-pinene (4.2%), (*Z*)-3-hexenyl acetate (3.97%), and acoradiene (3.8%) [29]. Flower extracts from seven varieties of flue-cured tobacco grown in China, obtained with different polar and non-polar organic solvents, showed a strong antifungal effect on *Valsa mali*, a microorganism causing rot disease of apple [30]. Flower-derived extracts had stronger antifungal activity than the respective leaf extracts, as they were richer in cembranoids, identified as the key effective antifungal components in the extracts. According to this study, the extracts obtained with non-polar or less-polar solvents (*n*-hexane, petroleum ether, ethyl acetate, dichloromethane) had a stronger inhibitory effect on the microorganism. The biological activities demonstrated by different fractions extracted from the flowers, leaves, and other tissues of *Nicotiana* species substantiated their potential in the development of biopesticides, in the isolation and genetic studies on plant defense, and other contemporary trends in bioengineering and molecular biology [6,19,30,31].

To the best of our knowledge, *Nicotiana* flowers have not been considered extensively as a source of obtaining aromatic products for cosmetic and perfumery use, although there is evidence for using *N. alata* and *N. suavolens* flower essential oils in perfumes (tobacco blossom and white flowers notes) [32]. On the other hand, *Nicotiana tabacum* flower oil and *Nicotiana tabacum* flower extract (names given according to the International Nomenclature of Cosmetic Ingredients, INCI) are referenced in the Cosmetic Ingredient Database, CosIng [33] as available cosmetic ingredients, with perfuming functions. *N. tabacum* flower oil and extracts are not listed in Annex II (banned cosmetic ingredients) or Annex III (restricted cosmetic ingredients) of Regulation No 1223/2009 (the Cosmetics Regulation) [34], unlike the case with nicotine as an individual substance. *N. tabacum* leaf essential oil and aromatic products (concrete, absolute) were not found to contain skin allergens, which are due to label indication, according to the provisions of the regulation [34,35,36]. The traditional natural aromatic products largely used in perfumery and cosmetics include plant essential oils (obtained by steam or hydrodistillation), concretes (obtained by extraction with non-polar organic solvents, further concentrated by complete removal of the solvent), resinoids (by extraction with polar solvents and concentration), absolutes (obtained by subsequent extraction of concretes and resinoids in ethanol and removal of the fraction that precipitates at cooling), pomades (obtained by hot or cold enfleurage of flowers), tinctures (ethanol or ethanol-water solutions of extracted plant materials or dissolved other extracts), and others [37].

As already stated, tobacco (*N. tabacum*) is an important industrial crop worldwide, providing large quantities of biomass. Tobacco flowers, however, have not been analyzed or processed, although the rationale for their utilization is sound. On one hand, they are a plant material with an abundance of fragrance and bioactive metabolites. On the other hand, the plants of broad-leaf tobaccos (Virginia, Burley, cigar, dark air, and fire-cured types) are topped, i.e., flower buds and some of the upper leaves are removed, in order to stimulate the development of the remaining lower leaves, thus generating discarded flower waste, rich in promising biochemicals [30,38]. In turn, the flowers of Oriental tobacco also represent unutilized waste from the agricultural production of cigarette tobacco, as they are left on the fields after leaf harvesting.

Bulgaria is one of the traditional producers of tobacco leaf, mainly of the export-oriented types Oriental (also referred to as “aromatic”, “Greek”, or “Turkish” tobacco) and Virginia flue-cured (Virginia bright) [39]. Cured and fermented leaf of the Oriental type has been commercially processed in the country since the 1960s to obtain tobacco aromatic products, concrete, resinoid, and absolute, for the fragrance and cigarette industries. In a series of previous studies, our research team investigated the phytochemical composition of the leaves of several *Nicotiana* species, *N. rustica*, *N. glutinosa*, and *N. alata*, experimentally grown on the fields of the Tobacco and Tobacco Products Institute, as well as the composition and properties of different aromatic products intended for phytopharmacy, cosmetic, and perfumery uses [40,41,42]. The results from these studies supported the potential of the species for larger-scale production and leaf processing into different final products.

Based on these considerations, this study aimed to determine the chemical composition (by GC-MS) and olfactory profiles of a certain type of an extraction aromatic product traditionally used in perfumery and cosmetics (flower concrete), obtained from the flowers of four *Nicotiana* species, in a direct comparison between them. We hypothesized that there would be significant differences in the extracted compounds between the species, thus producing flower concretes with specific individual features. We also hypothesized that the application of the selected flower processing and analytical approach would add new detail to the study of *Nicotiana* plants, as no previous data from this point of view was available. The outcomes from the investigation would provide evidence for the applicability of those products in perfumery and cosmetics, as well as an option for the utilization of a discarded plant material.

## 2. Results

Data about the moisture content of the initial plant material, fresh flowers of different *Nicotiana* species, as well as about the yield and appearance of the obtained concretes are presented in Table 1. The results suggested different concrete-yielding potentials of the species, with the clear differentiation of the common tobacco, *N. tabacum* (Virginia (FCV), and Oriental (OR) types. The yield was the lowest for *N. rustica*, probably related to the structure of the species’ flowers. Interestingly, the *N. alata* genotype with white flowers gave a low concrete yield, close to that of *N. rustica*, although *N. alata* is considered one of the most intensively scent-emitting species and is a popular ornamental plant [12,14,31]. The yield of concrete for the *N. alata* genotype with pink flowers, however, was higher, comparable with that of *N. glutinosa*. All extracted concretes had a similar texture and color, with waxy semi-solid yellow-green masses.

The olfactory profiles of the obtained concretes from fresh flowers of different *Nicotiana* species were individually assessed, and the resultant odor descriptions are given in Table 2.

As seen from the integrated odor descriptions, all *Nicotiana* flower concretes had a distinct perceptible odor, and differentiation of the odor profiles on a species and type basis was achieved.

**Figure 1:**
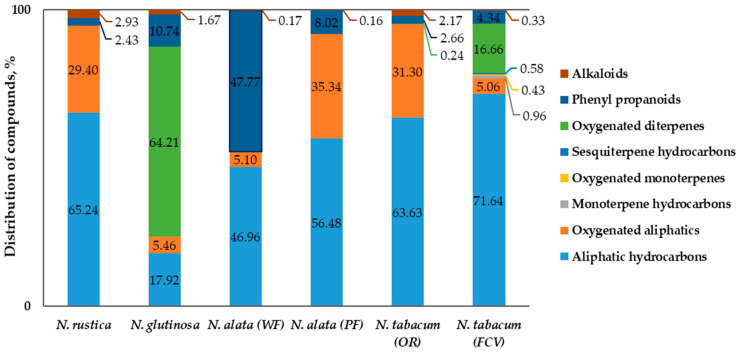
Profile of *Nicotiana* flower concretes (the sum of identified compounds = 100%); *N. alata (WF)*-genotype with white flowers; *N. alata (PF)*-genotype with pink flowers; *N. tabacum (OR)*-Oriental type; *N. tabacum (FCV)*-flue-cured Virginia type.

The results from the GC-MS analysis of the chemical composition of the flower concretes are presented in Table 3, and the distribution of the identified compounds by basic functional groups is shown in Figure 1.

Data revealed that the individual composition of the flower concretes varied considerably between the species; variations were observed both in terms of the number of identified compounds and major constituents (Table 3), as well as in the profile distribution of compounds (Figure 1).

In the concrete obtained from *N. rustica* flowers, 24 components (99.5% of the total content) were identified; only one of them had a concentration under 1% As seen from Table 3, the major constituents (over 3%) of the extract were: *n*-tetratriacontane (14.6%), *n*-triacontane (12.1%), pentanoic acid, 3-methyl (11.1%), tributyl acetylcitrate (9.1%), *n*-octacosane (5.5%), *n*-nonacosane (4.4%), *n*-dotriacontane (4.3%), *n*-hexatriacontane (3.7%), and (*Z*,*Z*)-linoleic acid (3.4%). The dominant group of substances in the concrete was that of aliphatic hydrocarbons (65.2%), and the rest of the identified components represented oxygenated aliphatics (29.4%), alkaloids (2.9%), and phenyl propanoids (2.4%).

In total, 31 components, equal to 97.8% of the total content, were identified in the concrete of *N. glutinosa* flowers; 15 of them were in concentrations over 1%, and the remaining 16 constituents were in concentrations under 1%. Seven components were identified as major (over 3%), as follows: Sclareol (25.9%), 3-α-hydroxy-manool (16.3%), 13-epimanool (14.9%), terephthalic acid, di(2-ethylhexyl) ester (9.5%), podocarpa-7-en-3-one, 13β-methyl-13-vinyl- (5.7%), *n*-nonacosane (3.5%), and *n*-tetratriacontane (3.5%) The profile of the concrete was dominated by the group of oxygenated diterpenes (64.2%), followed by aliphatic hydrocarbons (17.9%), phenylpropanoids (10.7%), oxygenated aliphatics (5.5%), and alkaloids (1.7%).

In total, 23 components, responsible for 97.3% of the total content, were identified in the concrete obtained from the flowers of the *N. alata* genotype with white flowers *(WF)*; 12 of them were in concentrations over 1% and the rest under 1% As seen from Table 3, seven were the major (over 3%) constituents of the concrete: Terephthalic acid, di(2-ethylhexyl) ester (42.9%), *n*-dotriacontane (11.9%), *n*-tetratriacontane (10.2%), *n*-triacontane (6.6%), *n*-octanoic acid (3.8%), phthalic acid, diisooctyl ester (3.6%), and *n*-tritriacontane (3.2%). The concrete composition was dominated by phenylpropanoids (47.8%) and aliphatic hydrocarbons (47.0%), followed by oxygenated aliphatics (5.1%) and alkaloids (0.2%).

In the second *N. alata* genotype *(PF)*, with pink flowers, 23 components (97.6%) were identified; 13 were in amounts over 1% and the remaining 10 constituents were in concentrations under 1%. Ten of the identified compounds were considered as major (over 3%): Tributyl acetylcitrate (30.7%), *n*-dotriacontane (14.7%), *n*-tetratriacontane (9.3%), *n*-triacontane (7.0%), *n*-tritriacontane (5.9%), terephthalic acid, di(2-ethylhexyl) ester (4.6%), *n*-hentriacontane (4.4%), *n*-nonacosane (4.2%), *n*-octanoic acid (3.4%), and phthalic acid, diisooctyl ester (3.3%). The profile of identified components constituted of aliphatic hydrocarbons (56.5%), followed by oxygenated aliphatics (35.3%), phenylpropanoids (8.0%), and alkaloids (0.2%).

The concrete of Oriental type tobacco flowers (*N. tabacum* (OR)) contained 25 identified components, representing 98.5% of the total content. Twenty-three of them were in concentrations over 1% and the rest were in concentrations under 1%. As seen from Table 3, the major constituents (over 3%) of the concrete were: *n*-tetratriacontane (15.0%), *n*-triacontane (13.3%), pentanoic acid, 3-methyl (12.2%), tributyl acetylcitrate (10.0%), *n*-octacosane (5.1%), *n*-nonacosane (3.8%), *n*-dotriacontane (3.2%), *n*-hexatriacontane (3.1%), *n*-pentacosane (3.1%), and (*Z*,*Z*)-linoleic acid (3.0%). The composition of the extract was dominated by aliphatic hydrocarbons (63.6%), followed by oxygenated aliphatics (31.3%), phenylpropanoids (2.7%), alkaloids (2.2%), and oxygenated diterpenes (0.2%).

As seen from Table 3, the biggest number of components, 45, was identified in the concrete obtained from the flowers of Virginia flue-cured tobacco (*N. tabacum* (FCV)); they were responsible for 95.03% of the total content. In total, 19 of them were in concentrations over 1% and the remaining 26 constituents were in concentrations under 1%. The major constituents (over 3%) of the extract were: *n*-hexacosane (12.9%), *n*-pentacosane (12.9%), *n*-tetracosane (8.3%), *n*-tricosane (8.1%), *n*-heptacosane (5.1%), duvatrienediol isomer (α) (4.9%), *n*-octacosane (4.3%), 4,8,13-duvatriene-1,3-diol isomer (α) (4.3%), *n*-triacontane (4.0%), (*Z*,*Z*)-linoleic acid (3.5%), and *n*-nonacosane (3.4%). The dominant compound group in the concrete was that of the aliphatic hydrocarbons (71.6%), followed by oxygenated diterpenes (14.7%), oxygenated aliphatics (5.1%), phenylpropanoids (4.3%), monoterpene hydrocarbons (0.96%), sesquiterpene hydrocarbons (0.6%), oxygenated monoterpenes (0.4%), and alkaloids (0.3%).

## 3. Discussion

The color of the extracted concretes, yellow-green, was related to the extraction of natural pigments and their derivatives, while the waxy texture was obviously due to the high proportions of extracted hydrocarbons, as seen from the GC-MS analysis. The yield of flower concretes, varying from 1.42% to 5.24%, could be considered satisfactory, as similar ranges were observed for other essential oil-bearing plants [43]. In particular, the yield of concrete from the Oriental type tobacco (3.14%) was higher, but consistent with the findings for the benzene and petroleum ether-extracted concrete yield from another Oriental variety (2.02%) [44]. Further comparison is hard to make, as to the best of our knowledge, these are the first results on the yield or description of flower concretes obtained from different *Nicotiana* species. As seen from Table 2, the odor intensity and the described olfactory notes of *Nicotiana* flower concretes suggested a specific contribution and good combination capacity from the perfumer’s point of view. The odor profile of *N. alata* concrete (both genotypes) was consistent with the descriptions of the essential oil isolated from the species, ‘summary hay and leather scent with dry floral tonality, very inviting’ [32]. The odor description of *N. tabacum* (OR) flower concrete in this study was more favorable than the assessment ‘indefinite but agreeable’ given by the above-cited study [44]. It should be noted that the odor profiles of the flower-derived concretes were completely different from those of the concretes obtained from the leaves of the same species [40,41,42], as well as from the typical ‘tobacco aroma’ (referred to the aroma of *N. tabacum* leaf) [9].

The peculiarities of the olfactory expression of *Nicotiana* flower concretes were related to the observed differences in their chemical composition. In order to emphasize the specificity of the obtained flower concretes, the results are discussed consecutively on a species (and genotype) basis below. Reasonably, data in this study varied considerably from previous findings, considering the fact that nearly all available references were about headspace flower volatiles, and not about volatiles extracted with non-polar solvents in a final ready-to-use aromatic product.

The major volatiles of *N. rustica* flowers, in a headspace analysis of scent emissions from the flowers of nine *Nicotiana* species [12], were benzaldehyde (64.9%), 1,3,3-trimethyl-7-oxabicyclo[4.1.0] heptan-2,5-dione (9.9%), benzyl alcohol (5.6%), and α-cedrene (4.2%), as well as nicotine (4.0%, at night and 21.4%, at day). In our study, terpenoids were not the major components of *N. rustica* flower concrete, which was dominated by aliphatic hydrocarbons and oxygenated aliphatics. Our results differed from the data in the study of the volatile flower oil of *N. rustica* [29], obtained by methylene chloride extraction followed by vacuum-steam distillation/*n*-hexane extraction, which identified as major components nicotine (25.9%), aromadendrene (11.4%), (*Z*)-3-hexenol (10.4%), decene (6.2%), eremophilene (5.2%), and methylheptanone/heptenol (3.6%). In our study, nicotine was also present in a considerable amount (2.9%), although significantly lower than the values cited above (25.9% [29] and 21.4% [12]). The presence of tributyl acetylcitrate as a major component in the concrete (9.1%), described with a “faint sweet herbaceous odor” [45], correlated with the recorded olfactory profile of the concrete. All those differences are explicable by not only the applied analytical approaches, as stated earlier, but also by genetic (variety) and cultivation-related differences.

A specific feature of the concrete of *N. glutinosa* flowers was the high content of labdane diterpenes (64.2% of the identified composition). As seen from Figure 1, the profile of *N. glutinosa* concrete differed specifically compared with the rest of the species in the study, being the only one dominated by oxygenated diterpenes. Those results are consistent with previous findings that labdane-type diterpenes are principal secondary metabolites in *N. glutinosa* [46,47], as well as with the existing data about the mechanism of transportation of metabolites involved in plant defense, such as alkaloids (nicotine), sclareol, manool, and other diterpenes, from the site of synthesis to the aerial organs of *Nicotiana* species [7,20,21]. The diterpene alcohol sclareol, C_20_H_36_O_2_, was the major diterpene (25.9% of TIC) in the flower concrete (Table 3); its odor is associated with sweet, balsamic, clary sage, amber, woody, and weedy notes [41]; therefore, the high concentration of sclareol corresponded well with the sensory description of the concrete, green, and slightly floral odor with honey-like undernotes. Besides sclareol, other diterpenes structurally related to ambergris odorous components were present, 13-epimanool (14.9%), 3-α-hydroxy manool (16.0%), and podocarp-7-en-3-one, 13β-methyl-13-vinyl- (5.7%). The content of sclareol in the flower-derived concrete (25.9%) was considerably higher than the levels established in the aromatic products obtained from the leaves of the same plants in our previous work, 3.6% in *N. glutinosa* leaf essential oil, 14.2% in leaf concrete, and 6.9% in leaf resinoid, respectively [41]. These results are in compliance with earlier findings about the distribution of the sclareol/13-epi-sclareol mixture within *N. glutinosa* plants [47], in particular that upper leaves and flower buds accumulated the highest amounts, up to 632 µg/g tissue. Therefore, our results about the composition of the obtained flower concrete, coupled with the relatively high yield (2.5%), provide additional arguments in favor of the commercial production and processing of the species, especially considering the importance of providing ambergris alternatives and diterpene-rich natural aromatic products for the fragrance industry, as discussed elsewhere [41].

The two *N. alata* genotypes in this study, one with white and one with pink flowers, revealed substantial differences with regard to the concrete composition, obviously genotype defined, as all plants were grown side-by-side and uniformly processed. The two genotypes of the species differed in terms of some individual compounds, most impressively in tributyl acetylcitrate (0.8% WF vs. 30.7% PF), and terephthalic acid, di(2-ethylhexyl) ester (42.9% WF vs. 4.6% PF) contents, as well as in the overall profile of the compounds, especially in the share of oxygenated aliphatics and phenylpropanoids. As those classes of volatile constituents are important odor contributors [37,45], the differences in chemical composition were perceived as odor details in the olfactory evaluation of the two genotypes as well (Table 2). Although further research is definitely needed, these first results suggested that the genotype should be taken into consideration when referring to *N. alata* flower (and leaf) extracts, as different odor contribution or biological activities might be expected.

Although parallels to previously published data might be biased, considering the intrinsic differences between headspace collections (from incised or living flowers) and processed flower products, as already stated, an interesting pattern was still noticeable in both *N. alata* flower concretes. Several studies highlighted that a characteristic monoterpene combination, consisting of 1,8-cineole, β-myrcene, limonene, sabinene, linalool, α- and β-pinene, and α-terpineol (“the cineole cassette”), was emitted by the flowers of many *Nicotiana* species [12,14,15,25,48]. For example, the major volatiles of the *N. alata* scent, in a fragrance chemistry study of different *Nicotiana* species [12], were 1,8-cineole (12.6–21.1%), linalool (10.0–27.5%), E-nerolidol (4.2–10.5%), E,E-farnesal (2.7–16.1%), and 3-methylbutyloxime (7.8–12.8%). Similar major volatiles were identified in another study on *N. alata* fragrance patterns [15], 1,8-cineole (29.2%), 3-methylbutyloxime (11.2–15.7%), E-nerolidiol (7.2%), E-4,8-dimethylnona-1,3,7-triene (6.3%), E-ꞵ-farnesene (3.3%), ꞵ-myrcene (3.1%), and sabinene (3.1%). In contrast to the above scent-related studies, those characteristic monoterpenes of the “cineole cassette” were not identified by us in the analyzed *N. alata* concretes, probably due to extraction, degradation, and other mechanisms, which is worthy of future investigation in order to understand the mechanisms involved better and to optimize the composition of the regarded concentrated ready-to-use extraction product.

As a general observation, the chemical composition of the concrete extracted from the flowers of the Oriental tobacco (*N. tabacum* (OR)) was comparable with that of *N. rustica* flower concrete (Table 3, Figure 1). The major volatiles were 3-methyl-pentanoic acid (3-methylvaleric acid, C_6_H_12_O_2_), described by a herbaceous, cheese-like, animal, and sharp odor, and tributyl acetylcitrate (C_20_H_34_O_8_), with a faint, sweet, and herbaceous odor. Those, together with other minor compounds, such as 3-methyl-butanoic acid (isovaleric acid, with cheesy, pungent, sweaty, stinky, feet, tropical odor, with ripe fatty and fruity notes) and 4-methyl-hexanoic acid (strong sour, cheesy odor), determined the olfactory profile of the concrete, and a very strong green odor with fresh undertones, reminiscent of freshly cut hay (Table 2). In fact, isovaleric acid and especially 3-methylvaleric acid are reported as the characteristic trait of highly aromatic Oriental tobacco cultivars, and are used as markers to discriminate between tobacco types and tobacco aromas [9]. The concrete was the only one, beside that of *N. glutinosa*, which contained sclareol, although in low amounts (0.2%), which is consistent with previous findings that Oriental and cigar type tobaccos are the only *N. tabacum* types capable of synthesizing both labdanoid and cembranoid diterpenes [9].

The concrete obtained from the flowers of the Virginia type tobacco, *N. tabacum* (FCV), had a more specific composition, in terms of the number of the identified individual components and the represented chemical classes (Table 3, Figure 1). It was the only product in this study that contained, although in minor amounts, monoterpene and sesquiterpene hydrocarbons and oxygenated monoterpenes, with a 0.96%, 0.58%, and 0.43% share, respectively. Additionally, only in this sample were the α- and β-isomers of duvatrienediol, a macrocyclic cembrene diterpene, identified, in a ratio of 2:1, which did not differ from the data at their first identification in the leaves of Burley tobacco in 1962 [49]. As stated earlier, Virginia type tobacco leaf contained only cembranoid, but not labdanoid, diterpenes [9,27], and our results were consistent with that; furthermore, our results corresponded to the findings that duvatrienediols were at much higher concentrations in younger upper plant organs [50]. The odor profile of the obtained flower concrete, a very strong green with slight floral and honey-like undertones, was obviously formed by a complex of major and minor components, as generally outlined for *Nicotiana* flower aroma [20,21,28]. Some of those odor-impact components might include linalool (with citrus, floral, sweet, rose, woody, green odor), linalyl acetate (with sweet, green, citrus, bergamot, floral, terpenic, spicy odor), solanone (with fruity, tobacco, melon odor), limonene (citrus, herbal, terpenic, camphoreous odor), syringaldehyde (plastic, woody, tonka, cocoa, sweet, creamy, nutty odor), and others [27,37]. In the discussion of the specific features of Virginia tobacco flower concrete, it should be outlined that the respective plants were the only ones in this study grown on a different large-scale production field, although in the same region, and the respective agricultural practices securing leaf yield and quality had been implemented by the farmer during plant vegetation. On the other hand, the rest of the species were planted in a field experiment, side-by-side, and developed under identical conditions. Therefore, some of the observed differences are associated to some extent with the influence of environmental and agro-technical factors, such as soil, terrain, fertilization and irrigation rates, plant protection, and others.

Certainly, an additional explanation for the above-discussed differences to previous findings could be found in the specific daily phenology and scent emission patterns observed for *Nicotiana* species. In general, most of the species, and especially those of the *Alatae* section, emitted considerably more scent, 9 ± 3 times, at night than during the day [12], but the patterns of diurnal vs. nocturnal emissions varied considerably among the species and classes of compounds [12,15,23,24]. *N. rustica*, in particular, emitted more nicotine (about five times) and nitrogenous compounds diurnally; the opposite was characteristic for *N. alata*, *N. bonariensis*, *N. longiflora*, and other species [12]. Monoterpene emissions, in turn, were higher at night, regardless of the species [12,15,23]. Although fresh flowers in this study were collected in a period of relatively higher aromatics levels [22], in the morning, the plant circadian rhythm might have an influence on the flower concrete yields and composition, which opens opportunities for future research.

Finally, in view of the possible use of the studied aromatic products in perfumery and cosmetics, two more points may be worth discussion. First, all concretes obtained from the flowers of the studied *Nicotiana* species, as the results in Table 3 suggested, contained nicotine, the typical native alkaloid of the genus. More significant nicotine concentrations were found in the concretes from *N. rustica*, *N. tabacum* (OR), and *N. glutinosa* flowers, but even the two genotypes of *N alata*, considered as the most low-nicotine species of the genus [42], produced nicotine in the concrete. In a brief comparison with other aromatic products from *Nicotiana* plants, the flower concretes in this study had higher nicotine concentrations than the respective flower [26,27] or leaf essential oils [40,41,42], obtained by distillation. Nicotine levels were higher than those found previously in the respective leaf-extracted concretes of *N. rustica* (0.32%) [40], and *N. glutinosa* (0.46%) [41] but comparable to *N. alata* leaf concretes (0.36% (WF) and 0.29% (PF)) [42]. On the other hand, the nicotine content in all flower concretes was significantly lower than that in the resinoids, another type of natural aromatic products, extracted from the leaves of the same species, from 32.92% (*N. glutinosa*) to 44.17% (*N. alata (PF)*) [40,41,42]. Therefore, the potential of *Nicotiana* flower concretes in perfumery and cosmetic formulations should be compliant with the specific functions assigned to any of them as an ingredient, as well as with the final concrete concentration in the completed product. The second aspect of the possible use of those natural aromatic products in skin-contacting perfumery and cosmetic products is the presence of human allergens. In this study, the obtained *Nicotiana* flower concretes did not contain any of the fragrance allergens, requiring obligatory indication in the list of ingredients of leave-on (above 0.001%) and rinse-off (above 0.01%) products [35,36], with the single exception of *N. tabacum (FCV)* flower concrete, which contained minor amounts of limonene (0.5%) and linalool (0.2%).

Thus, the conducted study demonstrates the potential of four *Nicotiana* species as aromatic plants. The accumulated new data about *Nicotiana* species, from a very specific aspect, namely the processing of fresh flowers into natural aromatic products with possible use in perfumery and cosmetics, added original details to the current knowledge about the genus. Furthermore, the results about the yield, olfactory profile, and chemical composition of the obtained flower concretes could provide new sources of interest for the fragrance industry. Additionally, a possible alternative use of a valuable, but discarded, plant material is suggested by the outcomes of this investigation.

## 4. Materials and Methods

### 4.1. Plant Material

**Figure 2:**
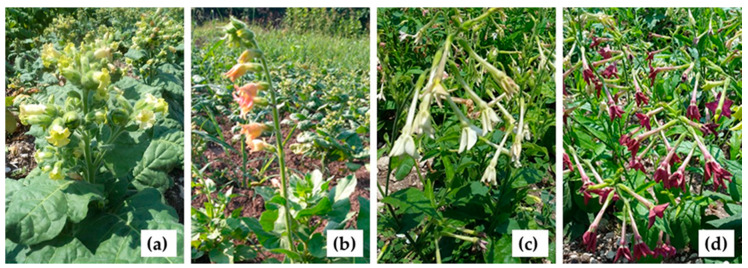
Flowers of *Nicotiana* plants at picking from the field: (**a**) *N. rustica*, (**b**) *N. glutinosa*, (**c**) *N. alata* genotype with white flowers, and (**d**) *N. alata* genotype with pink flowers (photos by authors).

The study was conducted with four different *Nicotiana* species, *N. rustica* L, *N. glutinosa* L, *N. alata* Link&Otto (represented by two individual genotypes, with white *(WF)* and with pink *(PF)* flowers), and *N. tabacum* (represented by two tobacco types, Oriental (OR) and Virginia (FCV)). The species were grown side-by-side in the fields of the Tobacco and Tobacco Products Institute-Bulgarian Agricultural Academy, situated in the region of Plovdiv, southern Bulgaria. The only exception were Virginia tobacco plants (*N. tabacum*), which were grown in a farmer’s field in the same region. All flowers were collected in June 2018, directly from the field (Figure 2; common tobacco flowers are not shown). Individual flowers were hand-picked in the morning, between 7 and 11 am, put in sterile tightly closed glass containers, and immediately processed. Before extraction, the samples, in amounts of approximately 0.500 kg, were carefully examined in order to eliminate the presence of impurities (such as unbloomed or overbloomed flowers, stems, soil particles, and others).

The moisture content of fresh flowers was determined by oven-drying to constant weight at 105 ± 1 °C [51]. All results, except for the concrete yield, were expressed on a dry weight (DW) basis.

### 4.2. Obtaining of Nicotiana Flower Concretes

The concretes from each of the species were obtained by twofold extraction with *n*-hexane (Sigma-Aldrich, St. Louis, MO, USA), in a batch mode, for 60 and 30 min at a temperature of 30 °C and raw material-to-solvent ratio of 1:10, followed by concentration on a rotary vacuum evaporator at a water bath temperature of 35 °C [43].

### 4.3. Olfactory Evaluation of the Concretes

The olfactory evaluation of the concretes was performed in a sensory laboratory [52] at the University of Food Technologies, Plovdiv, Bulgaria. Two professional perfumers and three aroma chemists (each having experience in the field >10 years; 4 females, one male) participated and evaluated the samples independently. At the time of the procedure, each of the panelists was in a normal healthy condition and mood. Three sniffs from the test strip were performed and the procedure was repeated three times daily within three days. The testers were asked to give their evaluation in the form of descriptive analysis; the individual responses were collected in paper ballots and then integrated to obtain the respective odor description [37].

### 4.4. GC-MS analysis of Nicotiana Flower Concretes

Concretes (50 µL) were diluted in 100 µL of pyridine (Sigma-Aldrich) and 100 µL of *N,O*-bis(trimethylsilyl)trifluoroacetamide (BSTFA; Supelco, Bellefonte, PA, USA), and incubated at 70 °C for 45 min. After derivatization, the samples were diluted with 150 µL of chloroform and injected (1 µL) in a system comprised of a 7890A gas chromatograph (Agilent Technologies Inc, Santa Clara, CA, USA) and a 5975C mass selective detector (Agilent Technologies Inc, Santa Clara, CA, USA). The column was HP-5 ms (30 m × 0.32 mm (i.d.); film thickness 0.25 μm), operated under the following conditions: Temperature increase from 40 (0 min) to 230 °C at 5 °C/min, held at 230 °C for 10 min; injector and detector temperatures of 250 °C; helium as a carrier gas at a 1 mL/min constant flow rate; mass detector scan range *m*/*z* = 50–550; split mode (5:1). Identification of the detected compounds was carried out using mass spectra library data ([53], NIST 08 database; own libraries). Calculation of the retention (Kovat′s) indices was done using a calibration mixture of *n*-alkanes (C_8_–C_40_) in *n*-hexane. The content of the identified compounds was presented as a percentage of the total ion current (TIC), following the normalization method of the recorded peak areas.

### 4.5. Statistics

All data were presented as mean value ± standard deviation, resulting from a threefold repetition of experiments. Statistical significance of differences was assessed by ANOVA and Tukey’s multiple comparison test (*p* < 0.05).

## 5. Conclusions

The GC-MS analysis of the concretes obtained through extraction with *n*-hexane from the fresh flowers of four *Nicotiana* species, *N. rustica*, *N. glutinosa*, *N. alata*, and *N. tabacum*, identified their major and minor constituents. Differences were observed between the species, as well as between the genotypes studied. The characteristic olfactory profiles of the concretes and their sufficient yields are a prerequisite for the possible processing of *Nicotiana* flowers and the obtaining of new aromatic products with use in perfumery and cosmetics. These are the first results, which characterize the flowers of the regarded *Nicotiana* species as potent sources for obtaining these natural aromatic products.

## Figures and Tables

**Table 1:** Basic indices of the plant material (*Nicotiana* flowers) and the obtained concretes.

Index	*N. rustica*	*N. glutinosa*	*N. alata (WF)* ^1^	*N. alata (PF)* ^2^	*N. tabacum (OR)* ^3^	*N. tabacum (FCV)* ^4^
Moisture (fresh flowers), %	86.61 ± 0.75 ^5^	81.13 ± 0.70	85.14 ± 0.75	84.64 ± 0.73	84.33 ± 0.73	84.49 ± 0.74
Yield of concrete, % FW ^6^	0.21 ± 0.01	0.48 ± 0.01	0.27 ± 0.01	0.38 ± 0.01	0.57 ± 0.01	0.81 ± 0.01
Yield of concrete, % DW ^7^	1.42 ± 0.01 ^a^	2.46 ± 0.02 ^b^	1.58 ± 0.01 ^a^	2.65 ± 0.02 ^b^	3.14 ± 0.03 ^c^	5.24 ± 0.05 ^d^
Appearance of concretes ^8^	Waxy, semi-solid masses with yellow-green color					

^1^*N. alata (WF)*-genotype with white flowers; ^2^*N. alata (PF)*-genotype with pink flowers; ^3^*N. tabacum (OR)*-Oriental type tobacco; ^4^*N. tabacum (FCV)*-flue-cured Virginia type tobacco; ^5^ data expressed as mean (*n* = 3) ± standard deviation; ^6^ FW—fresh weight; ^7^ DW-dry weight; ^8^ by visual assessment; ^a–d^ values with different superscripts within a row differed significantly (*p* < 0.05).

**Table 2:** Olfactory profiles of *Nicotiana* flower concretes.

Species	Odor Description ^1^
*N. rustica*	Typical green, herbaceous odor, with slight honey-like undertones
*N. glutinosa*	Green, slightly floral odor with honey-like undertones
*N. alata (WF)* ^2^	Floral, honey-like odor with sweet undertones
*N. alata (PF)* ^3^	Typical floral odor with slight green and honey-like undertones
*N. tabacum (OR)* ^4^	Very strong green odor with fresh undertones, reminiscent of freshly cut hay
*N. tabacum (FCV)* ^5^	Very strong green odor with slight floral and honey-like undertones

^1^ Integrated description (5-member panel, *n* = 9); ^2^ *N. alata (WF)*-genotype with white flowers; ^3^ *N. alata (PF)*-genotype with pink flowers; ^4^ *N. tabacum (OR)*-Oriental type tobacco; ^5^ *N. tabacum (FCV)*-flue-cured Virginia type tobacco.

**Table 3:** Volatile composition (GC-MS) of *Nicotiana* flower concretes.

No	Compounds	RI ^1^	Content, % of TIC ^2^					
			*N. rustica*	*N. glutinosa*	*N. alata (WF)* ^3^	*N. alata (PF)* ^4^	*N. tabacum (OR)* ^5^	*N. tabacum (FCV)* ^6^
1	Butanoic acid, 3-methyl-	851	1.71 ± 0.01 ^7^	0.25 ± 0.00	nd	nd	1.89 ± 0.01	nd
2	α-3 hexene	939	nd ^8^	nd	nd	nd	nd	0.13 ± 0.00
3	Pentanoic acid, 3-methyl	946	11.11 ± 0.10	0.19 ± 0.00	nd	nd	12.22 ± 0.11	nd
4	Benzaldehyde	965	nd	nd	nd	nd	nd	0.29 ± 0.00
5	β-Pinene	979	nd	nd	nd	nd	nd	0.11 ± 0.00
6	Hexanoic acid, 5-methyl-	983	nd	1.24 ± 0.01	nd	nd	nd	nd
7	Hexanoic acid, 4-methyl-	1008	2.27 ± 0.02	2.58 ± 0.02	nd	nd	2.49 ± 0.02	nd
8	Limonene	1030	nd	nd	nd	nd	nd	0.51 ± 0.00
9	1,8-cineole	1032	nd	nd	nd	nd	nd	0.15 ± 0.00
10	Linalool	1103	nd	nd	nd	nd	nd	0.17 ± 0.00
11	*n*-Octanoic acid	1173	nd	nd	3.81 ± 0.03	3.43 ± 0.03	nd	nd
12	Naphtalene	1181	nd	nd	nd	nd	nd	0.14 ± 0.00
13	Linalyl acetate	1259	nd	nd	nd	nd	nd	0.09 ± 0.00
14	2-Methylmaphtalene	1295	nd	nd	nd	nd	nd	0.21 ± 0.00
15	1-Methylnaphtalene	1312	nd	nd	nd	nd	nd	0.11 ± 0.00
16	Nicotine	1367	2.86 ± 0.02	1.63 ± 0.01	0.17 ± 0.00	0.16 ± 0.00	2.14 ± 0.02	0.31 ± 0.00
17	Solanone	1374	nd	nd	nd	nd	nd	0.48 ± 0.00
18	β-Caryophyllene	1419	nd	nd	nd	nd	nd	0.07 ± 0.00
19	β-Farnesene	1448	nd	nd	nd	nd	nd	0.08 ± 0.00
20	*n*-Pentadecane	1500	nd	nd	nd	nd	nd	1.55 ± 0.01
21	*n*-Hexadecane	1600	nd	nd	nd	nd	nd	2.41 ± 0.02
22	4-allyl-syringol	1614	nd	nd	nd	nd	nd	1.02 ± 0.01
23	4-propyl syringol	1620	nd	nd	nd	nd	nd	1.36 ± 0.01
24	Syringaldehyde	1673	nd	nd	nd	nd	nd	0.72 ± 0.00
25	*n*-Heptadecane	1700	nd	nd	nd	nd	nd	0.52 ± 0.00
26	Tetradecanoic acid	1778	nd	nd	nd	nd	nd	0.30 ± 0.00
27	Phenanthrene	1790	nd	nd	nd	nd	nd	0.17 ± 0.00
28	Athracene	1798	nd	nd	nd	nd	nd	0.11 ± 0.00
29	*n*-Octadecane	1800	nd	nd	nd	nd	nd	0.13 ± 0.00
30	Pentadecanoic acid	1875	nd	nd	nd	nd	nd	0.38 ± 0.00
31	*n*-Nonadecane	1900	nd	nd	nd	nd	nd	0.61 ± 0.00
32	Sclareoloxide	1906	nd	0.30 ± 0.00	nd	nd	nd	nd
33	*n*-Hexadecanoic acid	1979	1.08 ± 0.01	0.21 ± 0.00	0.14 ± 0.00	0.13 ± 0.00	1.19 ± 0.01	nd
34	13-Epimanool	2055	nd	14.95 ± 0.13	nd	nd	nd	nd
35	*n*-Eicosane	2000	nd	nd	nd	nd	nd	0.74 ± 0.00
36	Thunbergol	2065	nd	nd	nd	nd	nd	2.27 ± 0.02
37	*n*-Heneicosane	2100	1.63 ± 0.01	0.36 ± 0.00	1.13 ± 0.01	1.02 ± 0.01	1.79 ± 0.01	0.30 ± 0.00
38	Methyl octadecanoate	2128	nd	nd	nd	nd	nd	0.49 ± 0.00
39	Duvatrienediol isomer (α)	2163	nd	nd	nd	nd	nd	4.86 ± 0.04
40	(*Z*,*Z*)-Linoleic acid	2168	3.39 ± 0.03	0.56 ± 0.00	0.25 ± 0.00	0.23 ± 0.00	3.03 ± 0.02	3.52 ± 0.03
41	Octadecanoic acid	2188	nd	nd	nd	nd	nd	0.61 ± 0.00
42	*n*-Docosane	2200	0.25 ± 0.00	0.75 ± 0.00	0.43 ± 0.00	0.39 ± 0.00	0.27 ± 0.00	1.23 ± 0.01
43	Duvatrienediol isomer (β)	2214	nd	nd	nd	nd	nd	2.01 ± 0.01
44	Sclareol	2222	nd	25.89 ± 0.24	nd	nd	0.24 ± 0.00	nd
45	4,8,13-Duvatriene-1,3-diol isomer (α)	2275	nd	nd	nd	nd	nd	4.26 ± 0.03
46	4,8,13-Duvatriene-1,3-diol isomer (β)	2282	nd	nd	nd	nd	nd	2.43 ± 0.02
47	Tributyl acetylcitrate	2254	9.10 ± 0.08	0.31 ± 0.00	0.76 ± 0.00	30.69 ± 0.29	10.01 ± 0.09	nd
48	Podocarp-7-en-3-one, 13β-methyl-13-vinyl-	2274	nd	5.65 ± 0.05	nd	nd	nd	nd
49	3-α-Hydroxy manool	2286	nd	16.02 ± 0.15	nd	nd	nd	nd
50	*n*-Tricosane	2300	1.35 ± 0.01	0.37 ± 0.00	0.32 ± 0.00	0.29 ± 0.00	1.48 ± 0.01	8.07 ± 0.07
51	*n*-Tetracosane	2400	1.79 ± 0.01	0.48 ± 0.00	0.72 ± 0.00	0.65 ± 0.00	1.97 ± 0.01	8.28 ± 0.07
52	*n*-Pentacosane	2500	2.77 ± 0.02	0.33 ± 0.00	0.81 ± 0.00	0.73 ± 0.00	3.05 ± 0.02	12.86 ± 0.11
53	*n*-Hexacosane	2600	2.05 ± 0.01	0.46 ± 0.00	0.62 ± 0.00	0.56 ± 0.00	2.26 ± 0.01	14.13 ± 0.13
54	*n*-Heptacosane	2700	2.51 ± 0.02	0.59 ± 0.00	0.45 ± 0.00	0.40 ± 0.00	2.76 ± 0.02	5.14 ± 0.04
55	Phthalic acid, diisooctyl ester	2712	2.37 ± 0.02	1.03 ± 0.01	3.62 ± 0.03	3.26 ± 0.03	2.61 ± 0.02	nd
56	*n*-Octacosane	2800	5.52 ± 0.05	1.16 ± 0.01	0.82 ± 0.00	0.74 ± 0.00	5.07 ± 0.04	4.31 ± 0.03
57	Terephthalic acid, di(2-ethylhexyl) ester	2869	nd	9.48 ± 0.08	42.85 ± 0.40	4.56 ± 0.04	nd	nd
58	*n*-Nonacosane	2900	4.37 ± 0.04	3.49 ± 0.03	1.21 ± 0.01	4.09 ± 0.03	3.81 ± 0.03	3.36 ± 0.03
59	*n*-Triacontane	3000	12.07 ± 0.11	1.19 ± 0.01	6.62 ± 0.06	6.96 ± 0.05	13.28 ± 0.12	3.95 ± 0.03
60	*n*-Hentriacontane	3100	2.54 ± 0.02	0.90 ± 0.00	2.65 ± 0.02	4.38 ± 0.04	2.09 ± 0.02	nd
61	*n*-Dotriacontane	3200	4.31 ± 0.04	1.51 ± 0.01	11.92 ± 0.10	14.72 ± 0.13	3.15 ± 0.03	nd
62	*n*-Tritriacontane	3300	2.80 ± 0.02	0.84 ± 0.00	3.18 ± 0.03	5.86 ± 0.05	2.08 ± 0.02	nd
63	*n*-Tetratriacontane	3400	14.55 ± 0.13	3.49 ± 0.03	10.15 ± 0.09	9.13 ± 0.08	15.01 ± 0.14	nd
64	*n*-Heptatriacontane	3500	1.37 ± 0.01	1.16 ± 0.01	1.90 ± 0.01	2.71 ± 0.02	1.51 ± 0.01	nd
65	*n*-Hexatriacontane	3600	3.72 ± 0.03	0.45 ± 0.00	2.75 ± 0.02	2.47 ± 0.02	3.09 ± 0.02	nd
	Sum of the identified		97.49	97.82	97.28	97.56	98.49	95.03

^1^ RI-retention (Kovat’s) index; ^2^ identified at >0.05% of TIC; ^3^ *N alata (WF)*-genotype with white flowers; ^4^ *N alata (PF)*-genotype with pink flowers; ^5^ *N tabacum (OR)*-Oriental type; ^6^ *N tabacum (FCV)*-flue-cured Virginia type; ^7^ data expressed as mean (*n* = 3) ± standard deviation; ^8^ nd-not detected or <005% of TIC.
